# Isolation of Intestinal Parasites of Public Health Importance from Cockroaches (*Blattella germanica*) in Jimma Town, Southwestern Ethiopia

**DOI:** 10.1155/2014/186240

**Published:** 2014-02-04

**Authors:** Haji Hamu, Serkadis Debalke, Endalew Zemene, Belay Birlie, Zeleke Mekonnen, Delenasaw Yewhalaw

**Affiliations:** ^1^Department of Medical Laboratory Sciences and Pathology, College of Public Health and Medical Sciences, Jimma University, Jimma, Ethiopia; ^2^Department of Statistics, College of Natural Sciences, Jimma University, Jimma, Ethiopia; ^3^Department of Biology, College of Natural Sciences, Jimma University, Jimma, Ethiopia

## Abstract

Cockroaches are claimed to be mechanical transmitters of disease causing microorganisms such as intestinal parasites, bacteria, fungi, and viruses. This study assessed the potential of the German cockroach *Blattella germanica* in the mechanical transmission of intestinal parasites of public health importance. A total of 2010 cockroaches were collected from 404 households in Jimma Town, southwestern Ethiopia. All the collected cockroaches were identified to species as *B. germanica*. The contents of their gut and external body parts were examined for the presence of intestinal parasites. Overall, 152 (75.6%) of the 210 batches were found to harbor at least one species of human intestinal parasite. *Ascaris lumbricoides, Trichuris trichiura, Taenia spp, Strongyloides*-like parasite, *Entamoeba histolytica/dispar/moshkovski, Giardia duodenalis* and *Balantidium coli* were detected from gut contents. Moreover, parasites were also isolated from the external surface in 22 (10.95%) of the batches. There was significant difference in parasite carriage rate of the cockroaches among the study sites (*P* = 0.013). In conclusion, *B. germanica* was found to harbor intestinal parasites of public health importance. Hence, awareness on the potential role of cockroaches in the mechanical transmission of human intestinal parasites needs to be created. Moreover, further identification of the Strongyloides-like worm is required using molecular diagnostics.

## 1. Introduction

Cockroaches are distributed throughout the world and they are among the most notorious insects inhabiting apartments, food handling establishments, and health care facilities. Over 3500 species of cockroaches have been identified. Thirty of these species are more adapted to human habitation or synanthropic. Of these, *Blattella germanica* (German cockroach), *Periplaneta americana* (American cockroach), and *B. orientalis* (the Oriental cockroach) are considered the most common pests to humans [1–3].

Cockroaches have indiscriminate dietary habits. Moreover, adult cockroaches have the ability to survive without food for several weeks. They have the ability to breed all year long in suitable environmental conditions. These features of cockroaches, together with their nocturnal activity, probably make them widespread [1, 4].

Several evidences show that cockroaches are carriers of medically important parasites including helminths and protozoa [5–9]. For instance, a field survey carried out in 11 primary schools in Taiwan documented that 4% of the *P. americana* and 10% of *B. germanica* examined harbored cysts of *Entamoeba histolytica/dispar/moshkovskii* on their cuticle and/or in the digestive tract [10]. Besides parasites, several species of bacteria and fungi that can potentially cause human diseases have been isolated from cockroaches [3, 5, 11]. Such pathogenic microorganisms are isolated from cockroaches probably because cockroaches frequently feed on human feces. Apart from acting as mechanical carriers of microorganisms, cockroaches are the major sources of indoor allergens. Exposure and sensitization to cockroach allergen are associated with asthma-related health problems [12], the magnitude of which depends on race and socioeconomic status [13].

Intestinal parasites are relatively common among residents in Jimma Town [14]. Examination of soil around residential areas in the town also shows that these gastrointestinal parasites are very common [15], probably due to poor human excreta disposal mechanism. The warm and moist environmental condition of Jimma Town makes the area an ideal natural habitat for the inhabitation of cockroaches.

Despite the abundance of cockroaches in residential areas in Jimma Town and the high prevalence of intestinal parasites in this urban setting, to our knowledge, there is no documented data on the role of cockroaches as carriers of intestinal parasites in the study area. Lack of information on the role of cockroaches in carrying these human parasites is what initiated this study, which is aimed at isolating parasites of public health importance from cockroaches collected from residential houses. The findings of the study will shed light on the potential role of cockroaches in the mechanical transmission of intestinal parasites and help to design more efficient control intervention strategy for the control of synanthropic cockroaches.

## 2. Materials and Methods

### 2.1. Study Setting

The study was conducted in Jimma Town, southwestern Ethiopia, from February to March 2012.

The study area is located 335 kms southwest of Addis Ababa, at an average altitude of 1,780 meters above sea level. The town has a climatic condition locally known as “*Woynadega*” (1,500–2,400 m above sea level). The town is generally characterized by a warm climate with a mean annual maximum temperature of 30°C and a mean annual minimum temperature of 14°C.

The annual rainfall ranges from 1138 to 1690 mm. The maximum precipitation occurs during the months of June to September, with minimum rainfall between December and January. Humid and hot climate makes the area conducive for cockroaches.

### 2.2. Cockroach Sampling, Identification, and Parasite Isolation

Two thousand and ten cockroaches were collected over a period of two months. The cockroaches were collected from 404 households selected from five of the 13 *kebeles* (the smallest administrative units in Ethiopia). The five *kebeles* were selected randomly. Indoor collection of cockroaches was carried out from selected households two months prior to the indoor residual spraying (IRS) operation for malaria control in the *kebeles*. Only adult cockroaches with an intact body were processed in the laboratory. The cockroaches were collected using empty jars coated with a thin film of vaseline baited with a piece of bread soaked in water. The collection jars were put at 19:00 hr and retrieved at 7:00 hr in the morning.

The trapped cockroach specimens were placed in labeled jars and transported to the Medical Parasitology Laboratory, Jimma University, for identification and further processing.

Morphological identification of the cockroaches was carried out using standard taxonomic keys [16]. Two hundred and ten batches of cockroaches (each batch with 10 cockroaches) were processed. Internal (gut) and external body surface contents of the cockroach specimens were processed as described elsewhere (3). Parasite species isolated from cockroaches' gut and external body surfaces were identified following Cheesbrough [17].

### 2.3. Data Analysis

In order to gain insight of the data, exploratory data analysis was performed. Cross-tabulation of the outcome presence of intestinal parasites and covariates (*kebele* and cockroach body part) was performed. In order to test whether the observed counts differ from the expected counts, a Chi-square test was employed.

## 3. Results

A total of 2,010 cockroaches (201 batches) were collected indoors from 404 households selected from five *kebeles* of Jimma Town, southwestern Ethiopia. All the collected cockroaches were identified to species level as *B. germanica*. Of the 201 batches of cockroaches screened, 152 (75.6%) were found to harbor at least one intestinal parasite species. There was a significant difference (*P* = 0.013) in the prevalence of intestinal parasites recovered from the batches of cockroaches among different *kebeles* (Table 1). Moreover, there was a significant difference in helminths carriage of the cockroaches among *kebeles* (*P* = 0.032) and the difference in protozoa parasite carriage of cockroaches among *kebeles* was also highly significant (*P* < 0.001).

Parasites were identified from the internal contents (gut) of all the positive batches of the cockroaches. Of the 201 batches of cockroaches examined, 22 (10.9%) and 152 (75.6%) batches were found to harbor parasites on their external body surfaces and guts, respectively (Table 2).

With respect to the types of parasites isolated, overall, 40.3% (95% CI: 33.52–47.08) of the batches harbored helminths only, 4.98% (95% CI: 1.97–7.98) of them had only protozoa, and the remaining 30.35% (95% CI: 23.99–36.70) had both helminths and protozoa.

**Figure 1:**
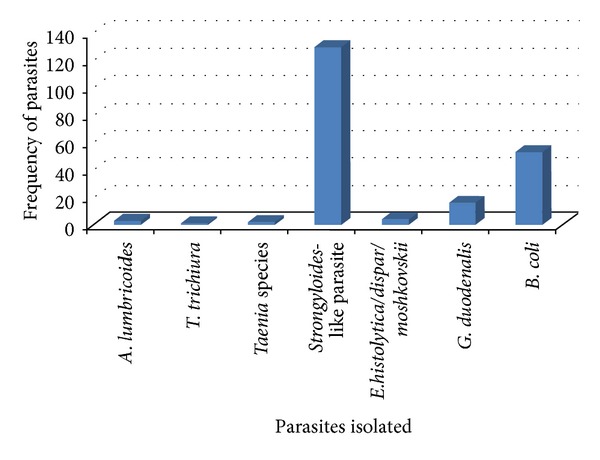
Percentage of parasite species isolated from populations of *B. germanica* in Jimma Town, southwestern Ethiopia, 2012.

Overall, seven species of medically important parasites were identified (Figure 1). The helminths include *A. lumbricoides*, *T. trichiura*, *Taenia* species, and an unidentified *Strongyloides*-like parasite. The protozoan parasites isolated were *E. histolytica/dispar/moshkovskii*, *G. duodenalis*, and *B. coli*. In this study, the most predominant parasite isolated was the suspected *Strongyloides*-like parasite.

## 4. Discussion

In this study, intestinal parasite species of medical importance were identified from the body surfaces and gut contents of the cockroaches. Accordingly, 75.6% of the batch of cockroach specimens examined had been found to harbor at least one species of human intestinal parasites.

A similar high parasite carriage rate (77.52%) had been reported from external body surfaces of cockroaches from Nigeria [8]. In contrast, no parasite species were isolated from cockroach specimens collected from residential areas; in Iran, however, in the same study, the percentage of cockroach specimens collected from public hospitals carrying intestinal parasites was low [3]. In Thailand, 54.1% of cockroach specimens collected from market places was reported to harbor parasite species [6]. Differences in the hygienic condition of the environments, including human excreta disposal, may account for the observed variation in the parasite carriage rate among different settings.

Overall, seven species of intestinal parasites were isolated from the cockroach specimens. The protozoan parasite species isolated were *E. histolytica/dispar/moshkovskii*, *G. duodenalis*, and *B. coli*. In a previous study, the potential role of cockroaches in the mechanical transmission of *E. histolytica* cysts had been reported [10]. Moreover, four species of intestinal helminthes, *A. lumbricoides*, *T. trichiura*, *Taenia* species, and an unidentified *Strongyloides*-like parasite, were isolated.

Several studies had previously reported that some of these parasite species and different species of bacteria and fungi were isolated from cockroaches [7, 8, 18]. Feeding starved roaches with microorganisms also resulted in the recovery of the microorganisms in the feces of the roaches [19, 20]. No oocysts of the coccidian parasites (*Cryptosporidium* and *Cyclospora* species) had been isolated from cockroach specimens in contrast to previous reports [6, 9].

## 5. Conclusions

Intestinal parasite carriage rate of cockroaches in Jimma Town was high. In this study, many parasite species which are known to cause intestinal parasitosis in humans had been isolated mainly from the gut contents of the cockroach specimens. The finding of this study sheds light on the potential role of cockroaches in the mechanical transmission of human intestinal parasites.

## Figures and Tables

**Table 1:** Percentage of parasites isolated from populations of *B. germanica *by *kebele *in Jimma Town, southwestern Ethiopia, 2012.

*Kebele *	Batch no. of cockroaches examined	Parasite isolated	*n* (%)	95% CI
Bossa Addis	32	Helminths	15 (46.88)	(29.58, 64.17)
		Protozoa	5 (15.63)	(3.04, 28.20)
		Total	**17 (53.13)**	**(35.83, 70.42)**

Ginjo	35	Helminths	26 (74.29)	(59.8, 88.77)
		Protozoa	8 (22.86)	(8.95, 36.77)
		Total	**26 (74.29)**	**(59.8, 88.77)**

Kochi	26	Helminths	19 (73.08)	(56.03, 90.13)
		Protozoa	5 (19.23)	(4.08, 34.38)
		Total	**19 (73.08)**	**(56.03, 90.13)**

Hermata Mentina	36	Helminths	28 (77.78)	(64.20, 91.36)
		Protozoa	13 (36.1)	(20.42, 51.80)
		Total	**29 (80.56)**	**(67.62, 93.48)**

Bocho Bore	72	Helminths	54 (75.00)	(65.00, 85.00)
		Protozoa	40 (55.56)	(44.08, 67.03)
		Total	**61 (84.72)**	**(76.41, 93.03)**

Total	201	Helminths and protozoa	152 (75.62)	(69.69, 81.57)

CI: confidence interval.

**Table 2:** Percentage of parasites isolated from the gut contents and external body surfaces of the populations of *B. germanica *in Jimma Town, southwestern Ethiopia, 2012.

Body part examined	Parasite isolated	*n* (%)	95% CI
External body	Only helminths	15 (7.46)	(3.83, 11.10)
	Only protozoa	5 (2.48)	(0.33, 4.64)
	Both	2 (0.10)	(0.00, 2.37)
	Total	**22 (10.95)**	**(6.63, 15.26)**

Gut	Only helminths	87 (43.28)	(36.43, 50.13)
	Only protozoa	55 (27.36)	(21.20, 0.34)
	Both	10 (4.98)	(1.96, 7.98)
	Total	**152 (75.62)**	**(69.69, 81.57)**

Total	Only helminths	81 (40.30)	(33.52, 47.08)
	Only protozoa	10 (4.98)	(1.97, 7.98)
	Both	61 (30.35)	(23.99, 36.70)
	Total	**152 (75.62) **	**(69.69, 81.57)**

CI: confidence interval.
